# Prime-Boost Vaccination With a Novel Hemagglutinin Protein Produced in Bacteria Induces Neutralizing Antibody Responses Against H5-Subtype Influenza Viruses in Commercial Chickens

**DOI:** 10.3389/fimmu.2019.02006

**Published:** 2019-09-04

**Authors:** Violetta Sączyńska, Agnieszka Romanik-Chruścielewska, Katarzyna Florys, Violetta Cecuda-Adamczewska, Natalia Łukasiewicz, Iwona Sokołowska, Małgorzata Kęsik-Brodacka, Grażyna Płucienniczak

**Affiliations:** ŁUKASIEWICZ Research Network-Institute of Biotechnology and Antibiotics, Warsaw, Poland

**Keywords:** influenza, H5-subtype influenza viruses, H5N1, hemagglutinin, recombinant vaccine, vaccination and immunization, chicken, poultry

## Abstract

The highly pathogenic (HP) avian influenza virus (AIV), H5N1 and reassortant H5-subtype HPAIVs, H5N2, H5N6, and H5N8, cause high mortality in domestic birds, resulting in economic losses in the poultry industry. H5N1 and H5N6 also pose significant public health risks and H5N1 viruses are a permanent pandemic threat. To control HPAIVs, eukaryotic expression systems have traditionally been exploited to produce vaccines based on hemagglutinin (HA), a protective viral antigen. In contrast, we used a bacterial expression system to produce vaccine targeting the HA protein. A fragment of the HA ectodomain from H5N1, with a multibasic cleavage site deletion, was expressed in *Escherichia coli*, refolded, and chromatographically purified from inclusion bodies. The resulting antigen, rH5-*E. coli*, was validated in terms of conformational integrity and oligomerization status. Previously, the protective efficacy of rH5-*E. coli* adjuvanted with aluminum hydroxide, has been positively verified by challenging the specific pathogen-free layer chickens with homologous and heterologous H5N1 HPAIVs. Protection was provided primarily by the H5 subtype-specific antibodies, as detected in the FluAC H5 test. The present studies were conducted to assess the performance of alum-adjuvanted rH5-*E. coli* in commercial birds. Broiler chickens were vaccinated twice with 25 μg of rH5-*E. coli* at 2- and 4-week intervals, while the layer chickens were vaccinated with 5- to 25-μg antigen doses at 4- and 6-week intervals. Post-vaccination sera were analyzed for anti-H5 HA antibodies, using homologous ELISA and heterologous FluAC H5 and hemagglutination inhibition (HI) tests. Prime-boost immunizations with rH5-*E. coli* elicited H5 HA-specific antibodies in all the chickens tested. Two antigen doses administered at 4- or 6-week intervals were sufficient to induce neutralizing antibodies against H5-subtype HAs; however, they were ineffective when applied with a 2-week delay. In the layers, 80% to 100% of individuals developed antibodies that were active in the FluAC H5 and/or HI tests. A dose-sparing effect was seen when using the longer prime-boost interval. In the broiler chickens, 62.5% positivity was achieved in the FluAC H5 and/or HI tests. The trials confirmed the vaccine potential of rH5-*E. coli* and provided indications for anti-influenza vaccination with respect to the chicken type and immunization scheme.

## Introduction

The highly pathogenic (HP) avian influenza virus (AIV), H5N1, first emerged in China in 1996 and subsequently spread to Asia, Europe, the Middle East, and Africa (1). In Asia and Africa, H5N1 HPAIVs have become enzootic. After ongoing evolution, they are now grouped under different genetic clades. The evolution and spread of these viruses have been accompanied by frequent avian flu outbreaks in poultry, causing up to 100% mortality and resulting in enormous losses to the poultry industry (2). H5N1 HPAIVs continue to cause outbreaks in poultry and sporadic infections in humans (1). As of December 2018, a total of 860 laboratory-confirmed human cases of H5N1 influenza, including 454 fatalities, have been reported (3). In addition, H5N1 HPAIVs may acquire human-to-human transmission ability, and therefore, they pose a pandemic threat.

During the evolution of H5N1 HPAIVs, substantial reassortment events occurred, involving gene exchange between viruses, beginning with the generation of an H5N5 reassortant virus in 2008 (2). This has led to the creation of a range of H5Nx reassortants, such as H5N2, H5N6, and H5N8 HPAIVs, that have acquired novel neuraminidase proteins. H5N6 HPAIV was of particular concern in 2014, as it caused multiple outbreaks in Southeast Asia and a human infection (4). Moreover, in 2014, H5N8 and H5N2 HPAIVs spread rapidly and globally, substantially affecting many populations of domestic birds, owing to infection or mass culling (5, 6). H5N2, H5N6, and H5N8 HPAIV outbreaks continue to be reported in different regions of the word (1). These viruses pose a threat to avian health and H5N6 HPAIV is also a threat to human health.

Formerly, HPAIV infection in poultry had been controlled by eradication when outbreaks occur locally (7). Since H5N1 HPAIV from Asia has now spread and become enzootic, some countries have implemented AIV vaccination programs. Two disparate approaches have developed for using vaccines to control AIV (8). One is to eradicate AIV with no or very limited vaccination, while the other is to live with the virus in poultry, using vaccination to reduce production losses. Accordingly, preventive, emergency, or routine vaccinations are applied in the field (7). There are pros and cons of each approach (8). However, good quality vaccines are a critical tool for minimizing losses and help to reduce the spread of the virus. The majority of vaccines against H5N1 HPAIV have been used in a few enzootic countries where vaccination programs are directed to all poultry (9). Less than 1% of all vaccines have been administered in a focused, risk-based approach in other countries or regions. The current vaccines against AIV for poultry predominantly comprise oil-emulsified, inactivated whole AIV vaccines (~95%), but also live vectored vaccines. Concomitant with increasing use, alternative platforms for the production of anti-AIV vaccines have been explored. They involve manufacturing recombinant subunit vaccines, based on hemagglutinin (HA), the surface glycoprotein of influenza viruses that is capable of eliciting neutralizing antibodies (10). As a result, anti-H5N1 virus vaccines, such as Volvac® B.E.S.T AI + ND for chickens (11) and Panblok for humans (12) have been produced. An important feature of HA-based vaccines is their compatibility with the differentiating infected from vaccinated animals (DIVA) strategy (13). This strategy can be achieved by the detection of antibodies against neuraminidase of the specified subtype.

HA is initially synthesized as a single polypeptide chain, known as precursor HA (HA0). It contains a signal sequence and two subunits separated by a proteinase cleavage site, forming a prominent surface loop (14). HA0 exists as a single continuous polypeptide in a non-fusogenic conformation. Cleavage of viral HA produces structural rearrangements of the protein, which affect its low-pH fusogenic transition. Accordingly, the cleaved protein contains HA1 and HA2 subunits, connected by a disulfide bond and adopts a metastable, prefusion conformation, becoming fusogenic at low pH. Posttranslational cleavage of HA0 facilitates the fusion of viral and endosomal membranes. The cleavability of HA0 and the distribution of HA-activating proteases in the host are recognized as major virulence factors [for review, see (15)]. The low pathogenic (LP) and HP influenza viruses are characterized by having HAs with monobasic and multibasic cleavage sites, which are recognized by trypsin- and subtilisin-like proteases, respectively. The cleavage site structures of HPIVs have evolved by successive insertions of several basic amino acids, such as lysine and arginine.

Biosynthesis of HA during viral multiplication in the host cells, is a complex, co- and post-translational process, accompanied by intracellular transport (16, 17). The protein undergoes glycosylation, which affects its folding and oligomerization and the stability of the trimer (17). Therefore, eukaryotic expression systems are widely recognized as the preferred platforms for obtaining HA antigens for vaccine production. Mammalian (18–21), insect (18, 22–24), and yeast (25, 26) cells have been exploited for many years to produce HAs from various subtypes. In eukaryotic cells, full-length HAs have been successfully produced (22, 23). The target antigens for eukaryotic expression have typically been proteins, based on the entire (18, 24–26) or truncated (19–21) HA ectodomain. Of note, the expression and processing of HA proteins of different lengths in eukaryotes has proceeded under the same conditions as biosynthesis of the viral antigen.

Prokaryotes are not considered suitable for the production of transmembrane proteins or proteins that require post-translational modifications to gain native protein structure and function. These characteristics are true of influenza virus HA, which is an integral membrane glycoprotein with a large hydrophilic domain on the external surface of the membrane, a small hydrophobic transmembrane domain, and a hydrophilic cytoplasmic domain (27). Nevertheless, since 2007, attempts have been made to obtain protective antigens in prokaryotes [for review, see (28)]. These attempts were justified in view of the concomitantly published results showing considerable independence of HA folding from its glycosylation states (21, 29). To decrease surface hydrophobicity, various fragments of native HA antigens, devoid of the transmembrane domain, were expressed in bacteria, with or without the signal sequences (28). Until now, the vast majority of these so-called bacterial HAs have been based on the HA1 subunit expressed separately or as a component of conjugate vaccines. Only a few reports on ectodomain- and HA2 subunit-based bacterial HAs have been presented. As a rule, shorter fragments require more careful selection of peptides from the protein sequence. Designs of short HA fragments are often preceded by analyses of HA structure *in silico*. Thus far, bacterial HAs have mostly been expressed in the form of inclusion bodies (IBs), which promote high-yield protein production. Obtaining the vaccine antigen using this technique requires the solubilization of isolated IBs in denaturing buffer, HA protein refolding, and protein purification. Results published thus far show that not all bacterial HAs adopt the correct conformation, which is highly dependent on the HA fragment chosen for expression (30–32) and the refolding method (33). The development of a suitable refolding method is challenging. Truncated proteins used in bacterial expression systems lack at least some of the native HA regions that participate in antigen folding (signal sequence and anchoring peptide) and trimerization (HA2 subunit) during biosynthesis (27).

A variety of influenza virus HA antigens have been obtained using recombinant DNA technology. They have varied in length (full-length, based on the HA1 and HA2 subunits or the HA ectodomain) and oligomerization state (monomeric or at least partly oligomeric). As they are derived from bacterial, mammalian, insect, or yeast expression systems, the proteins have different glycosylation status (non-glycosylated or glycosylated) and glycosylation patterns (mammalian, insect, or yeast). Independently of their origin, recombinant HAs have been extensively studied for structure, the ability to bind to sialic acid-containing receptors, antigenicity, immunogenicity, and efficacy in challenge experiments and/or clinical trials. Since Th- and B-cell-dependent activities are effective or even sufficient for the control and resolution of influenza virus infection [for review, see (34)], pre-challenge testing of vaccine efficacy involves the measurement of anti-HA antibody responses. In particular, immunization studies have focused on the ability of the HA protein to induce serum antibodies that inhibit hemagglutination and/or neutralize influenza viruses *in vitro*. Titers of these antibodies measured in hemagglutination inhibition (HI) and/or virus microneutralization (VN) tests are widely considered as correlates of protection against influenza (35). Nevertheless, anti-influenza virus immunity in the absence of detectable HI (36) or HI and VN (37) antibodies has been reported. Therefore, there are growing calls to redefine the correlates of protection against influenza (35, 38–40).

Our previous (41) work and the work presented here respond to the demand for an inexpensive, safe vaccine against HPAIVs that can be used in preventive or emergency vaccinations in compliance with the DIVA strategy. A novel, ectodomain-based HA protein, with the sequence of the H5N1 HP viral strain, was expressed in *Escherichia coli*, refolded, and chromatographically purified from IBs. The antigen (amino acids [aa] 17–522, ΔRRRKKR), referred to as rH5-*E. coli*, was found to display characteristics of viral HA. Therefore, bacterial HA can be a valuable vaccine antigen when rationally designed and subjected to appropriate folding and purification methods.

Previously, we confirmed the protective efficacy of rH5-*E. coli* by means of experimental infections performed in specific pathogen-free (SPF) layer chickens (41). Prime-boost immunizations with rH5-*E. coli* (25 μg per dose) and aluminum hydroxide (alum) adjuvant at 4-week intervals protected 100% and 70% of chickens against challenge with homologous and heterologous H5N1 HPAIVs, respectively. Moreover, vaccination eliminated or delayed contact transmission of the homologous and heterologous viruses, respectively, and reduced virus shedding. Serological analyses performed in the course of challenge experiments indicated that rH5-*E. coli*-induced protection of chickens against viral challenge was primarily provided by antibodies detected in the ID Screen Influenza H5 Antibody Competition test (FluAC H5, IDVet) and not, if at all, by antibodies inhibiting hemagglutination. Thus, our previous work showed the role of H5 subtype-specific, non-HI neutralizing antibodies in conferring immunity against H5N1 HPAIVs. Consistently, we identified a novel and specific correlate of vaccine-induced protection and an accompanying test for pre-challenge evaluation of anti-H5N1 virus vaccines. As previously suggested, identification of epitopes, targeted by antibodies active in the FluAC H5 test, would be useful for the design of vaccines against H5N1 HPAIVs and perhaps other H5Nx viruses. The importance of identifying neutralizing epitopes in influenza virus HAs, for the development of influenza vaccines and therapeutics, is evident in view of recent reports (42, 43).

In this study, we examined the vaccine efficacy of rH5-*E. coli* in the two main types of chicken breeds, layer and broiler, under semi-field conditions, using different antigen doses and/or prime-boost intervals. The vaccine potential of our bacterial HA was confirmed in layer chickens. These birds responded to the prime-boost vaccination with alum-adjuvanted rH5-*E. coli* by vigorous production of anti-H5 HA antibodies that were active in indirect ELISAs (H5 iELISA), HI assays with H5N2 LPAIV, and FluAC H5 tests. Here, a dose-sparing effect was achieved. Broiler chickens developed much weaker immune responses compared to the layer chickens due to lower immunocompetence. Our immunization studies using rH5-*E. coli* and the reference H5 HA antigen, showed that the time interval between antigen doses was important for vaccination outcome. In general, this work provides clear indications for vaccination against influenza viruses with respect to chicken type and immunization scheme.

## Materials and Methods

### Production of H5 Hemagglutinin Antigen in *Escherichia coli* (rH5-*E. coli*)

The pIGCmT7Kes expression plasmid (44) containing cDNA encoding a fragment (aa 17–522, Δ341–346) of the H5 HA (pIGKesHA17522Δ) was obtained as described previously (41). The source sequence originated from the H5N1 A/swan/Poland 305-135V08-2006 strain of HPAIV (EpiFlu Database Accession No. EPI156789) and is listed in Table 1. For expression, the pIGKesHA17522Δ plasmid was transformed into the BL21 (DE3) *E. coli* strain. Glycerol stocks of the transformed bacteria cells were stored at −70°C.

**Table 1:** Antigens and antisera used in this study.

**Name**	**HA protein**	**Relevant AIV strain**	**Application**	**Origin**
rH5-*E. coli*	aa 17–522 ΔRRRKKR	A/swan/Poland/305-135V08/2006(H5N1)	Vaccine antigen tested in broiler (Exp 1) and layer chickens (Exp 2)	IBA
rH5-BEVS	aa 17–530 ΔRRRKKR 6x His	A/swan/Poland/305-135V08/2006(H5N1)	Reference antigen in rH5-*E. coli* analyses and broiler chicken vaccinations (Exp 1); antigen in H5 iELISA	OET
rH5-mammalian	aa 17–530 ΔRRRKKR 6x His	A/Bar-headed Goose/Qinghai/12/05(H5N1)	Reference antigen in rH5-*E. coli* analyses	ITC
H5N2 LPAIV	Full-length	A/turkey/Italy/80(H5N2)	Antigen in the HI test	x-OvO Ltd. IZSVe
Anti-H5N2 LPAIV antiserum	Full-length	A/turkey/Italy/80(H5N2)	Positive control in the HI test	x-OvO Ltd. IZSVe
Anti-H7N4 LPAIV antiserum	Full-length	A/mallard/Italy/4810-79/04(H7N4)	Negative control in the HI test	x-OvO Ltd. IZSVe
Anti-H7N7 LPAIV antiserum	Full-length	A/macaw/England/626/80(H7N7)	Negative control in the HI test	x-OvO Ltd. IZSVe

*Recombinant H5 hemagglutinin antigens, rH5-E. coli, rH5-BEVS, and rH5-mammalian, were produced in bacterial, baculoviral, and mammalian expression systems at the Institute of Biotechnology and Antibiotics (IBA; Warsaw, Poland), Oxford Expression Technologies Ltd. (OET) and Immune Technology Corporation (ITC), respectively. The low-pathogenic avian influenza virus (LPAIV) and anti-LPAIV antisera, certified by Istituto Zooprofilattico Sperimentale delle Venezie (IZSVe), were purchased from x-OvO Ltd*.

The target protein was expressed, refolded, and purified according to the quality-focused procedure for laboratory scale production, as described previously (41). Briefly, transformed BL21 (DE3) *E. coli* cells were cultured in LB medium with chloramphenicol and IPTG induction. The expressed protein was recovered by isolation of IBs, followed by solubilization of the protein under denaturing conditions. The protein solution was subjected to sequential purification on a DEAE Sepharose Fast Flow column (GE Healthcare, Uppsala, Sweden) and refolding by dilution and purification on a Phenyl Sepharose 6 Fast Flow (High-Sub) column (GE Healthcare). Finally, the antigen was formulated in 40 mM Tris-HCl, pH 8.0, with the addition of a protease inhibitor cocktail (Sigma-Aldrich, St. Louis, MO, USA), filtered through a 0.2-μm filter, and stored in aliquots at 4°C. The H5 HA protein thus obtained was subsequently referred to as rH5-*E. coli*.

### Analyses of rH5-*E. coli*

The protein concentration in samples collected at successive steps of the procedure was determined by the Bradford method. HA protein expression and purification were assessed by sodium dodecyl sulfate-polyacrylamide gel electrophoresis (SDS-PAGE), as described previously (41). The quantitative composition of the final rH5-*E. coli* preparations was determined by on-chip separation and detection in an Agilent 2100 Bioanalyzer (Agilent Technologies, Santa Clara, CA, USA) using the Agilent High Sensitivity Protein 250 kit. In this way, the purity of the different HA protein batches was estimated to range from 75 to 80%. The molecular mass of rH5-*E. coli* was calculated from the amino acid composition using GPMAW 8.2 software (Lighthouse, Odense, Denmark) and determined using a matrix-assisted laser desorption ionization time-of-flight (MALDI-TOF/TOF) mass spectrometer (4800 Plus, AB SCIEX, USA).

The antigenicity of rH5-*E. coli* was assessed by ELISA, using nine monoclonal (US Biological, Salem, MA, USA; Thermo Scientific, Waltham, MA, USA; Acris Antibodies, Herford, Germany) and two polyclonal (Immune Technology Corp., New York, NY, USA) antibodies. Antibodies were tested for activity against commercially available recombinant H5 HA proteins. According to previously published results (41, 45), these antibodies are suitable for vaccine antigen examination in terms of conformational integrity and the presence of epitopes targeted by H5 subtype-specific HI and VN antibodies. The sialic acid-binding activity and oligomerization status of rH5-*E. coli* were assessed using a hemagglutination test. ELISA and hemagglutination tests were performed as described previously (41).

### Reference H5 Hemagglutinin Antigens (rH5-BEVS and rH5-Mammalian)

Two H5 HA reference antigens were employed in the present study. The first one, referred to as rH5-BEVS, contained the full-length ectodomain of HA (aa 17–530). It was produced with the cleavage site deletion, ΔRRRKKR and a 6x-His tag at the C-terminus, using a baculovirus expression vector system (BEVS; Oxford Expression Technologies Ltd., Oxford, UK). The sequence of rH5-BEVS originated from the A/swan/Poland/305-135V08/2006(H5N1) strain of HPAIV, which is the same strain as rH5-*E. coli*. The second reference antigen, also a His-tagged H5 HA antigen, is referred to as rH5-mammalian. It comprised the 17–530-aa H5 HA fragment with the ΔRRRKKR deletion, the same as rH5-BEVS. It was produced in a mammalian expression system with a sequence derived from the A/Bar-headed Goose/Qinghai/12/05(H5N1) viral strain (Immune Technology Corp.). rH5-mammalian was highly homologous with both rH5-BEVS and rH5-*E. coli*. The reference antigens were specified as purified to at least 95%. rH5-BEVS and rH5-mammalian served as the reference antigens in the analyses of rH5-*E. coli*. rH5-BEVS was also used for chicken vaccination and the detection of anti-H5 HA antibodies in indirect ELISAs. The details of the reference antigens are summarized in Table 1.

### Chicken Vaccination and Sample Collection

Broiler and layer chickens (Ross 308 and Rossa 1 lines, respectively) were purchased from a commercial breeder on the day of hatching. They were maintained at an experimental poultry house with wheat straw beeding at a stock density of 5 birds/m^2^. Vaccines dedicated for commercial flocks were not administered. rH5-*E. coli* was examined for vaccination efficacy in two independent experiments (Exp 1 and Exp 2). In Exp 1, rH5-BEVS was used as the reference antigen. In both experiments, chickens were immunized twice with equal doses of the same HA antigen and alum adjuvant (1.3% Alhydrogel; Brenntag Biosector, Frederikssund, Denmark). The vaccine was administered in a volume of 200 μL by subcutaneous injection in the neck.

In Exp 1, four groups of 1-week-old broiler chickens (7 or 8 birds/group) were vaccinated in parallel with 25-μg doses of rH5-*E. coli* or rH5-BEVS. A booster was administered 2 or 4 weeks after priming. In Exp 2, eight groups of 3-week-old layer chickens (10 birds/group) were vaccinated with 25-, 15-, 10-, and 5-μg doses of rH5-*E. coli* at 4-week or 6-week intervals. The controls for Exp 1 and Exp 2 were non-vaccinated broiler and layer chickens (15 birds/group), respectively. Blood samples were taken from the wing veins, once or twice after both the prime and the boost, in the vaccinated groups and from the control chickens at the same time points. Blood samples were allowed to coagulate and were then centrifuged to collect serum, which was stored in aliquots at −70°C until assayed.

The test vaccine groups were denoted according to the chicken type (broiler, B or layer, L), the test or reference antigen dose (25 or 25_ref, 15, 10, or 5) and the time interval between doses (2, 4, and 6). The control groups for the vaccinated broiler and layer chickens in Exp 1 and Exp 2 were denoted B-controls and L-controls, respectively. The schemes of vaccination and blood collection are shown in Table 2.

**Table 2:** Experimental vaccination schemes.

**Exp**	**Chicken type**	**Antigen**	**Group**		**Dose**	**Age at priming**	**Prime-boost interval**	**Blood collection**	
			**Name**	**Size**				**post-prime**	**post-boost**
Exp 1	Broiler	rH5-*E. coli*	B-25/2	8	25 μg	1 week	2 weeks	2 weeks	2 weeks
			B-25/4	8	25 μg	1 week	4 weeks	2 weeks /4 weeks	1 week/2 weeks
	Broiler	rH5-BEVS	B-25_ref/2	7	25 μg	1 week	2 weeks	2 weeks	2 weeks
			B-25_ref/4	8	25 μg	1 week	4 weeks	2 weeks /4 weeks	1 week/2 weeks
	Broiler	None	B-controls	15	n/a	n/a	n/a	2 weeks 2 weeks /4 weeks	2 weeks 1 week/2 weeks
Exp 2	Layer	rH5-*E. coli*	L-25/4	10	25 μg	3 weeks	4 weeks	4 weeks	1 week/2 weeks
			L-15/4	10	15 μg	3 weeks	4 weeks	4 weeks	1 week/2 weeks
			L-10/4	10	10 μg	3 weeks	4 weeks	4 weeks	1 week/2 weeks
			L-5/4	10	5 μg	3 weeks	4 weeks	4 weeks	1 week/2 weeks
			L-25/6	10	25 μg	3 weeks	6 weeks	6 weeks	1 week/2 weeks
			L-15/6	10	15 μg	3 weeks	6 weeks	6 weeks	1 week/2 weeks
			L-10/6	10	10 μg	3 weeks	6 weeks	6 weeks	1 week/2 weeks
			L-5/6	10	5 μg	3 weeks	6 weeks	6 weeks	1 week/2 weeks
	Layer	None	L-controls	15	n/a	n/a	n/a	4 weeks 6 weeks	1 week/2 weeks

*The data for the tested (rH5-E. coli) and reference (rH5-BEVS) antigens are summarized in Table 1. The test vaccine groups were denoted according to chicken type (broiler, B or layer, L), the test or reference antigen dose in μg (25 or 25_ref, 15, 10, or 5), and the time interval between doses in weeks (2, 4, or 6). The control groups for vaccination studies in broiler chickens in Exp 1 and layer chickens in Exp 2 were denoted B-controls and L-controls, respectively. Details on the vaccination scheme and sample collection are provided in the Materials and Methods section*.

### Indirect and Competitive ELISAs (H5 iELISA and FluAC H5)

An H5 iELISA for the detection of anti-H5 HA IgY antibodies was performed using MediSorp plates (Nunc, Roskilde, Denmark) coated by overnight incubation at 2–8°C with 3 μg/mL rH5-BEVS in PBS. The coated plates were blocked with a Protein-Free T20 (PBS) Blocking Buffer (Pierce/Thermo Scientific). Sera from vaccinated and non-vaccinated chickens were individually diluted 1:200 (one-dilution H5 iELISA). To determine the endpoint titers, sera collected from each of the test and control chicken groups at the selected sampling times were pooled and then 2-fold serially diluted. All serum dilutions were performed in 2% BSA in a 0.05 M phosphate buffer containing 0.297 M NaCl and 0.0027 M KCl at pH 7.4. The diluted sera were applied to the antigen-coated wells and also to the non-coated wells as a control for non-specific binding. The dilution buffer was used as the blank control. The plates with test and control samples were incubated overnight at 2–8°C. Antigen-bound antibodies were detected using HRP-labeled, goat anti-chicken IgY (Fc-specific) antibodies (Pierce/Thermo Scientific). The plates were then incubated with secondary antibodies, diluted 1:13,000 with 2% BSA in standard phosphate buffered saline (PBS), for 1 h at 37°C. Reactions were developed with TMB (Sigma-Aldrich) at room temperature for 30 min and were subsequently stopped by the addition of 0.5 M H_2_SO_4_. Absorption was measured at 450 nm using a Synergy HT multi-detection microplate reader (BioTek Instruments Inc., Winooski, VT, USA). The mean absorbance values for blank control samples were subtracted from test samples.

The one-dilution H5 iELISA was performed for sera collected at each sampling time point, as indicated in Table 2. Samples were considered positive for anti-H5 HA IgY antibodies if the absorbance readings were more than 2 standard deviations above the mean absorbance values of the samples from the respective control chickens (cut-off value). Endpoint titers were determined for each of the pooled sera collected after administration of the boost dose (Table 2). The endpoint titer was defined as the highest dilution of the vaccinated chicken sera producing an absorbance value 4-fold higher than the absorbance value of the control chicken group.

The competitive ELISA, ID Screen Influenza H5 Antibody Competition-FluAC H5 (IDVet, Grables, France), which detects H5-subtype-specific antibodies in bird sera, was performed according to the manufacturer's instructions. Post-prime and/or post-boost sera were analyzed following the protocol, which improves the detection and sensitivity of the test and is suitable for all birds, except geese. The sample/negative control absorbance ratio of test samples was calculated and expressed as a competition percentage. Samples presenting a competition percentage ≥40%, between 35 and 40%, or ≤ 35% were considered negative, doubtful, or positive for the presence of anti-H5 HA antibodies, respectively.

### Hemagglutinin Inhibition (HI) Test

An HI test was conducted according to the OIE Manual of Diagnostic Tests and Vaccines for Terrestrial Animals (46). Erythrocytes used in the test were from SPF chickens (National Veterinary Research Institute, Puławy, Poland). Chicken sera were analyzed with the heterologous A/turk/Italy/80(H5N2) LPAIV strain at an HI unit (HIU) of 1:8. Each assay included control erythrocytes and control antisera. Antiserum against H5N2 LPAIV was used as a positive control, while anti-H7N4 and/or anti-H7N7 LPAIV antisera were used as negative controls. The viral antigen and antisera used in the HI test were purchased from x-OvO, Ltd. (Dunfermline, UK) and were certified by Istituto Zooprofilattico Sperimentale delle Venezie (Legnaro, Italy). Details are provided in Table 1.

Assays were performed in V-bottom 96-well plates (CellStar/Greiner Bio-One, Frickenhausen, Germany). Test and control sera were serially diluted 2-fold in Dulbecco's PBS (DPBS, Sigma-Aldrich) and then incubated for 25 min with 4 hemagglutination units (HAU) of H5N2 LPAIV. Simultaneously, a 1:4 dilution of sample not containing the viral antigen was incubated to provide the erythrocyte control. Next, a 1% erythrocyte suspension was added. Results were recorded after incubation for a minimum of 30 min.

HI activity was determined for sera collected at each of the sampling time points indicated in Table 2. Sera from vaccinated chickens were tested in parallel with those from non-vaccinated chickens. The HI titer was defined as the reciprocal of the highest dilution of serum that caused an inhibition of hemagglutination activity with 4 HAU of the inactivated antigen. Serum HI titers equal to or >1:16 were considered positive, while sera with titers of 1:8 or with undetectable antibodies were considered negative. For the vaccinated, layer chicken groups, the geometric mean titers (GMT) of HI-positive sera collected one and two weeks after the boost were calculated. For each of the test vaccine groups, the GMT of HI antibodies was calculated based on the highest titers determined for individual chickens, including those that were both HI-positive and -negative in the test. Data analysis was performed using Statistica software (StatSoft, Cracow, Poland). The Kruskal-Wallis and Mann-Whitney U non-parametric tests were used for the comparison of multiple and two groups, respectively. A value of *p* < 0.05 was considered significant.

### Ethics Statement

The experiments in this study were approved by the Second Local Ethical Committee for Animal Experiments at the Medical University of Warsaw (Poland), Permit Number 17/2009. All efforts were made to minimize suffering of the animals.

## Results

### Vaccine Antigen (rH5-*E. coli*)

The influenza vaccine antigen produced in bacteria encompasses aa 17–522 of HA from the H5N1 virus strain (A/swan/Poland/305-135V08/2006[H5N1]). It was a soluble, ectodomain-based protein truncated after the bromelain cleavage site. In addition to the ectodomain truncation, the basic amino acids, lysine (K) and arginine (R), were deleted from the cleavage site between the HA1 and HA2 subunits of the source H5 HA. The resulting antigen (aa 17-522, ΔRRRKKR), rH5-*E. coli* (Table 1), exclusively has the native HA sequence and does not contain any glycan structures. Independent of protease activity and buffer conditions, the protein will exclusively form a single continuous polypeptide. It was assumed that the correctly folded antigen would have a non-fusogenic conformation. Schematic representations of the source and the target H5 HA proteins can be found on the Biomedical Advances website (47).

rH5-*E. coli* was efficiently expressed in the *E. coli* BL21(DE3) strain transformed with the pIGCmT7Kes plasmid (44), harboring codon-optimized cDNA encoding the relevant H5 HA protein. The antigen was recovered in the form of insoluble, mis-folded IBs. This implied that suitable procedures would need to be developed to obtain a novel bacterial HA that possesses viral antigen characteristics. In practice, several refolding and purification methods were tested. For each method, the resulting antigen properties were assessed by mass spectrometry, ELISAs, and hemagglutination tests. The progress of purification was followed by separation and detection of the proteins using SDS-PAGE and an Agilent Bioanalyzer. Finally, we developed a refolding and purification protocol for the laboratory-scale production of the protein. The protocol focused on the quality and purity of the vaccine candidate. Details of the rH5-*E. coli* production procedure are provided in our previous paper (41). Briefly, the isolated IBs were solubilized and then subjected sequentially to purification on a DEAE-Sepharose column, refolding by dilution, and purification on a Phenyl-Sepharose column.

Each batch of rH5-*E. coli* used in vaccine formulation was validated. Protein analyses were performed in parallel with reference antigens of eukaryotic expression system origin (Table 1). These reference antigens were ectodomain-based H5 HA proteins (aa 17–530, ΔRRRKKR, 6x His) produced using a mammalian cell expression system (rH5-mammalian) and a baculovirus expression vector system (rH5-BEVS). The molecular mass of bacterial HA, as determined by mass spectrometry, was ~57 kDa. This was consistent with the value calculated based on the amino acid composition. Antigenicity tests showed that rH5-*E-coli* preserved the epitopes for the H5-subtype specific, HI and VN antibodies. The hemagglutination test confirmed both the correct conformation of the purified antigen and its ability to form functional oligomers. These results were consistent with previously presented data (41). Altogether, our studies showed that rH5-*E. coli* displays characteristics of the viral HA, which is a prerequisite for an effective vaccine antigen against influenza.

Immunization studies were performed in broiler chickens using rH5-BEVS (aa 17-530, ΔRRRKKR, 6x His) as the reference vaccine antigen (Table 1). The sequence of this antigen originated from the A/swan/Poland/305-135V08/2006(H5N1) strain of HPAIV, which is the same strain as rH5-*E. coli*. The determined molecular mass of rH5-BEVS was ~64 kDa. This differed from the theoretical value by ~6 kDa due to glycosylation. As for rH5-*E. coli*, rH5-BEVS was analyzed for antigenicity by ELISA and hemagglutination tests, prior to its use in vaccine formulation. According to data provided previously (41), this antigen was correctly folded and existed in part as a functional oligomer.

### Rationale to Experimental Design

Domestic birds are preferred when testing anti-HPAIV vaccines, since they are the natural host for AIVs and the main targets for vaccination. Currently, the only reliable way to evaluate how a vaccine will perform against a specific challenge virus is to conduct *in vivo* challenge trials in the target species (8). In line with this, the vaccine potential of our bacterial HA has been verified in experimental infection studies in chickens (41). However, in those studies, SPF layer chickens were vaccinated under laboratory conditions. Vaccines usually perform better in the laboratory than in the field due to the cleaner conditions and often because birds used experimentally are specific pathogen free and have not been exposed to other respiratory or immunosuppressive agents (8). This justifies the present immunization studies, which were conducted in chickens under standard rearing conditions. In terms of experimental design, the diversity of chicken breeds has been taken into account by using the Ross 308 and Rossa 1 lines, representing the broiler and layer hens, respectively. These two main types of chickens have been produced as a result of intensive genetic selection for improved feed conversion and rapid rate of growth or egg production (48). Broiler and layer chickens differ considerably in their growth and development, metabolism, body weight gain, and lifespan. Moreover, some data indicate that there is a relationship between performance traits and immunological characteristics in these chickens (49). In view of the above, different vaccine performance in broiler and layer chickens was anticipated.

To perform immunizations, the test and reference H5 HA antigens, rH5-*E. coli* and rH5-BEVS (Table 1), were formulated in an aluminum hydroxide adjuvant. Aluminum compounds are widely approved and cost-effective adjuvants that are known to be strong inducers of humoral immune responses, but poor inducers of cellular immune response (50). The vaccines in the current study were administered to broiler and layer chickens twice by subcutaneous injection of equal antigen doses. Similar to the challenge experiments, relatively high (25-μg) doses of alum-adjuvanted rH5-*E. coli* or rH5-BEVS were used with an inter-dose delay of 4 weeks. However, here, the study was expanded to include 2- and 6-week intervals between the prime and boost immunizations. Moreover, 15, 10, and 5 μg doses of rH5-*E. coli* were tested to estimate the minimum effective dose of antigen. Thus, another purpose of these studies was to determine the most effective vaccination strategy for the broiler and layer chickens, based on the prime-boost time interval and/or antigen dosage.

The short life expectancy of broiler chickens in commodity production (about 6 weeks) was considered when designing immunization studies in Exp 1. To include an inter-dose delay of 4 weeks, the first dose of antigens had to be given 1 week after hatching. Consequently, the chickens vaccinated at 2-week intervals for comparison were primed at the same age. Broiler chickens were divided into four test vaccine groups, each comprising eight or, exceptionally, seven chickens (Table 2). Two groups were vaccinated twice at 2- and 4-week intervals, with 25 μg of alum-adjuvanted rH5-*E. coli* (B-25/2 and B-25/4), while the other two were vaccinated with alum-adjuvanted rH5-BEVS (B-25_ref/2 and B-25_ref/4). The control group (B-controls) comprised fifteen, non-vaccinated broiler chickens.

Immunization studies in layer chickens in Exp 2 were designed using the scheme that was initially optimized in broiler chickens. It also included immunization protocols with longer prime-boost intervals and lower doses of the test antigen than those of Exp 1. In commodity production, layer chickens live for ~2 years. Thus, the prime immunizations could be performed in fully immunologically mature, 3-week-old birds to accomplish the designed vaccination schemes. Layer chickens were divided into eight test vaccine groups, each comprising ten chickens (Table 2). Four groups were vaccinated twice with 25, 15, 10, or 5 μg of alum-adjuvanted rH5-*E. coli* at a 4-week interval (L-25/4, L-15/4, L-10/4, and L-5/4). The remaining four groups were vaccinated with antigen doses of 25, 15, 10, or 5 μg at a 6-week interval (L-25/6, L-15/6, L-10/6, and L-5/6). The control group (L-controls) comprised fifteen, non-vaccinated chickens.

During the experimental time-course, blood samples for serological analyses were collected (Table 2). In the test vaccine groups, samples were collected once or twice after both the prime and the boost. The sampling time points were the same in the control chickens. Sera from vaccinated and control chickens were assayed for the presence of anti-H5 HA antibodies using ELISA and HI tests.

Successful vaccinal response in a flock is often monitored by demonstrating a rise in antibody titer within a few days of vaccination (51). This is why an iELISA, commonly used to assay humoral responses, was developed by our group to measure serum IgY antibodies against H5 HA in the vaccinated chickens. In the H5 iELISA, the homologous H5 HA antigen, rH5-BEVS, was used (Table 1). The one-dilution H5 iELISA enabled the assessment of each chicken as positive or negative for anti-HA antibodies and the determination of positivity rates in the test vaccine groups. The level of antibodies induced using a particular vaccination scheme was evaluated by endpoint titer determinations in pooled sera. As the minimum protective ELISA antibody levels for AIV have not been established (8), the results from the H5 iELISA could not be interpreted in terms of protection against influenza. Instead, they were treated as a basic indicator of chicken immune responses and immunogenicity of the tested or reference antigen.

In addition to the homologous H5 iELISA, the competitive ELISA test, FluAC H5 (IDVet), was used to detect H5-subtype-specific antibodies in chicken sera. FluAC H5 is a valuable screening tool for the diagnosis of H5-subtype influenza virus infections in birds, including H5N1 HPAIV infection. The competition percentages determined by the test, enabled the assessment of each chicken as negative, doubtful, or positive for the presence of anti-H5 HA antibodies and the calculation of positivity rates in the test vaccine groups. Initially, the results of the FluAC H5 test were treated as yet another indicator of the development of anti-HA antibody responses in chickens. However, in view of challenge experiments showing the role of H5-subtype-specific antibodies in conferring anti-influenza virus immunity, the results of the FluAC H5 test were reinterpreted. Accordingly, positivity in the FluAC H5 test here, is considered a specific correlate of vaccine-induced protection.

Additional serological analyses were performed using HI tests, which detect antibodies that block the binding of viruses to cellular receptors. These tests are typically used for influenza diagnostics and vaccine serology. In the assay performed here, the heterologous LPAIV strain, H5N2, was used as the antigen (Table 1). A search using the BLAST algorithm found that the HA of this virus strain and the vaccine antigens shared 90% sequence identity at aa 17–340, forming their HA1 subunits with epitopes for HI antibodies. Serum HI titers equal to or greater than 1:16 were considered positive in this study. On this basis, each chicken was scored as positive or negative. The positivity rates in each group at different time points and the GMT values for the respective HI-positive sera were calculated. In addition, the highest titers in the HI-positive and HI-negative chickens (HI titers ≤ 1:8) in each group were selected as the most representative for the group. These data were used to calculate the GMT of HI antibodies induced in chickens with a specified vaccination scheme and statistical differences between the test vaccine groups were evaluated. Consistent with widely accepted views, positivity in the HI test was regarded as an indicator of a protective immune response.

Results from serological analyses of samples collected during immunization studies were summarized by the classification of chickens as H5 iELISA positive, HI and/or FluAC H5 positive, or HI and FluAC H5 negative. Based on the percentage of chickens in these specified categories in each group, immune response patterns were generated to indicate the most effective vaccination strategies for commercial chickens.

**Figure 1:**
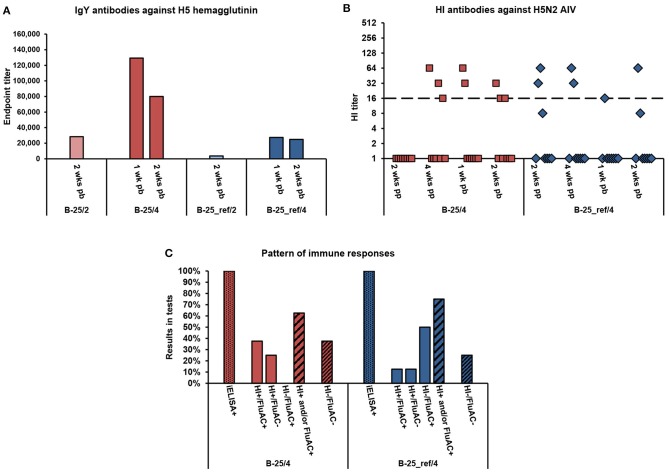
Serum antibody responses of broiler chickens to vaccination with alum-adjuvanted rH5-*E. coli* or rH5-BEVS. Four groups of 1-week-old broiler chickens (8 or 7 per group) in Exp 1 were vaccinated twice at 2- and 4-week intervals, with 25 μg of rH5-*E. coli* (B-25/2 and B-25/4) or rH5-BEVS (B-25_ref/2 and B-25_ref/4) and aluminum hydroxide (alum) adjuvant. Sera collected post-prime (pp) and/or post-boost (pb) were analyzed for the presence of anti-H5 HA antibodies using the indirect and competitive ELISAs, H5 iELISA and FluAC H5, respectively, and a hemagglutination inhibition (HI) assay with H5N2 low-pathogenic (LP) AIV. Tests were performed and interpreted as described in the Materials and Methods section. In **(A)**, the results for all of the test vaccine groups are presented. In **(B,C)**, only the results for chickens vaccinated at a 4-week interval are shown. The chickens vaccinated at a 2-week interval were seronegative in relevant tests (Table 3). **(A)** Endpoint titers of anti-H5 HA IgY antibodies in post-boost sera. Each bar represents the result for the pooled sera collected from one group of chickens at the indicated time point. **(B)** Titers of HI antibodies against H5N2 LPAIV in post-prime and post-boost sera. Each symbol represents the result for one chicken at the specified time point. A value of 1.0 indicates that the HI antibody titer was below the detection limit (1:8). The dashed line indicates the cut-off value in the HI test (1:16). **(C)** Pattern of immune responses to the prime-boost vaccination. Considering the results from all of the post-vaccination sera, chickens were classified as positive (+) or negative (–) in H5 iELISAs (iELISA) or HI and FluAC H5 tests (HI/FluAC). Each bar represents a percentage of the specified category of chickens in each group.

**Figure 2:**
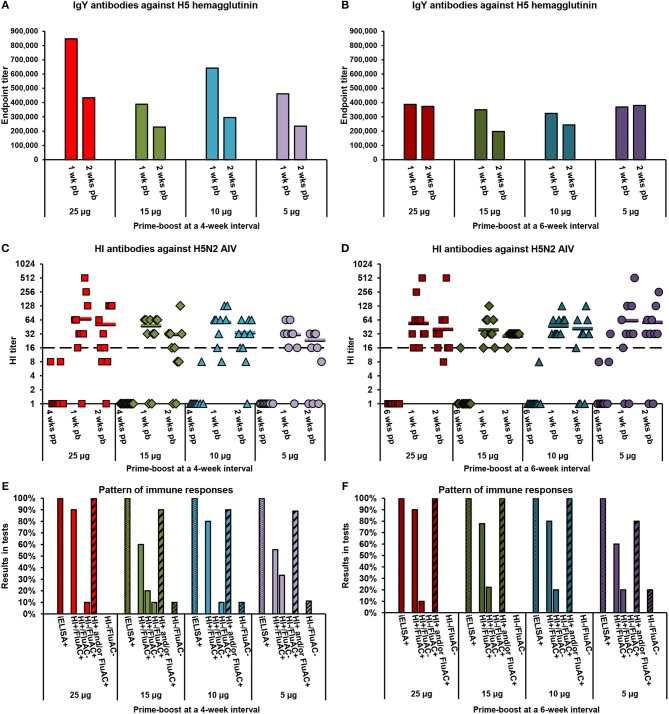
Serum antibody responses of layer chickens to vaccination with alum-adjuvanted rH5-*E. coli*. Eight groups of 3-week-old layer chickens (10 per group) in Exp 2 were vaccinated twice at 4-week **(A,C,E)** or 6-week **(B,D,F)** intervals with 25, 15, 10, or 5 μg of rH5-*E. coli* and aluminum hydroxide (alum) adjuvant. Sera collected post-prime (pp) and/or post-boost (pb) were analyzed for the presence of anti-H5 HA antibodies using the indirect and competitive ELISAs, H5 iELISA and FluAC H5, respectively, and a hemagglutination inhibition (HI) assay with H5N2 low-pathogenic (LP) AIV. Tests were performed and interpreted as described in the Materials and Methods section. **(A,B)** Endpoint titers of anti-H5 HA IgY antibodies in post-boost sera from chickens vaccinated at **(A)** 4-week and **(B)** 6-week intervals. Each bar represents the result for pooled sera collected from one group of chickens at the indicated time point. **(C,D)** Titers of HI antibodies against H5N2 LPAIV in post-prime and post-boost sera from chickens vaccinated at **(C)** 4-week and **(D)** 6-week intervals. Each symbol represents the result for one chicken at the specified time point. A value of 1.0 indicates that the HI antibody titer was below the detection limit (1:8). The horizontal lines are the geometric mean titers (GMT) of HI-positive sera (≥1:16), detected 1 or 2 weeks post-boost in particular groups of chickens. The dashed line indicates the cut-off value in the HI test (1:16). **(E,F)** Pattern of immune responses to the prime-boost vaccination at **(E)** 4-week and **(F)** 6-week intervals. Considering the results from all of the post-vaccination sera, chickens were classified as positive (+) or negative (−) in H5 iELISA (iELISA) or HI and FluAC H5 tests (HI/FluAC). Each bar represents a percentage of the specified category of chickens in each group.

Seropositivity results for anti-H5 HA antibodies in broiler and layer chickens vaccinated with rH5-*E. coli* and/or rH5-BEVS are presented in Tables 3, 4, respectively. Titers of anti-H5 HA IgY and HI antibodies in the post-prime and/or post-boost sera and the patterns of immune responses to vaccination in the broiler and layer chickens are shown in Figures 1A–C and Figures 2A–F, respectively. The representative HI titers and GMT values for the test vaccine groups are presented in Figures S1, S2, while results from statistical analysis of the HI assay data are in Tables S1, S2.

**Table 3:** Seropositivity for anti-H5 HA antibodies in broiler chickens vaccinated with alum-adjuvanted rH5-*E. coli* or rH5-BEVS.

**Group**	**Positivity in tests [%]**											
	**2 weeks** **post-prime**		**4 weeks** **post-prime**		**1 week** **post-boost**				**2 weeks** **post-boost**			
	**iELISA**	**HI**	**iELISA**	**HI**	**iELISA**	**FluAC**	**FluAC**	**HI**	**iELISA**	**FluAC**	**FluAC**	**HI**
	**+**	**+**	**+**	**+**	**+**	**+**	**+/–**	**+**	**+**	**+**	**+/–**	**+**
B-25/2^#^	100^#^	0^#^	n/a	n/a	n.d.	n.d.	n.d.	n.d.	100	0 ^..^	0 ^..^	0^#^
B-25/4^#^	87.5^#^	0^#^	50^#^	37.5^#^	100^#^	25^#^	12.5^#^	25^#^	100^#^	33	33	37.5^#^
B-25_ref/2*	86*	0*	n/a	n/a	n.d.	n.d.	n.d.	n.d.	86*	0*	0*	0*
B-25_ref/4^#^	100^#^	25^#^	50^#^	25^#^	100^#^	50^#^	0^#^	12.5^#^	100^#^	50^#^	25^#^	12.5^#^

Four groups of 1-week-old broiler chickens in Exp 1 were vaccinated twice at 2- and 4-week intervals with 25 μg of the test (rH5-E. coli) or reference (rH5-BEVS) antigens and aluminum hydroxide (alum) adjuvant. The test vaccine groups were denoted according to the chicken type (B, broiler), the tested or reference antigen dose in μg (25 or 25_ref), and the time interval between doses in weeks (2 or 4). Post-vaccination sera were analyzed for the presence of anti-H5 HA antibodies using the indirect and competitive ELISAs, H5 iELISA and FluAC H5, respectively, and a hemagglutination inhibition (HI) assay with H5N2 low-pathogenic (LP) AIV. Tests were performed and interpreted as described in the Materials and Methods section. Data are presented as the percentage of chickens positive (+) in iELISA and HI tests, and positive (+) or doubtful (+/−) in the FluAC test at the indicated sampling time points. The #, ^*^,”, and ^..^ symbols following the seropositivity percentages denote that 8, 7, 6, or 5 chickens were tested, respectively, in the group comprising 8 (#) or 7 (^*^) chickens.

**Table 4:** Seropositivity for anti-H5 HA antibodies in layer chickens vaccinated with alum-adjuvanted rH5-*E. coli*.

**Group**	**Positivity in tests [%]**											
	**4**^****a****^ **or 6**^****b****^ **weeks post-prime**				**1 week post-boost**				**2 weeks post-boost**			
	**iELISA**	**FluAC**	**FluAC**	**HI**	**iELISA**	**FluAC**	**FluAC**	**HI**	**iELISA**	**FluAC**	**FluAC**	**HI**
	**+**	**+**	**+/-**	**+**	**+**	**+**	**+/-**	**+**	**+**	**+**	**+/-**	**+**
L-25/4 ^x^	100 ^ax^	0 ^ax^	0 ^ax^	0 ^a^^∧^	100 ^x^	100 ^x^	0 ^x^	90 ^x^	100 ^x^	89 ^∧^	0 ^∧^	70 ^x^
L-15/4 ^x^	60 ^ax^	0 ^ax^	0 ^ax^	0 ^ax^	100 ^x^	70 ^x^	0 ^x^	80 ^x^	100 ^x^	63 ^#^	0 ^#^	60 ^x^
L-10/4 ^x^	90 ^ax^	0 ^ax^	0 ^ax^	0 ^a^^∧^	100 ^x^	90 ^x^	0 ^x^	89 ^∧^	100 ^x^	90 ^x^	0 ^x^	80 ^x^
L-5/4 ^x^	90 ^ax^	0 ^ax^	0 ^ax^	0 ^a^^∧^	100 ^x^	50 ^x^	0 ^x^	89 ^∧^	100 ^∧^	40 ^x^	10 ^x^	56 ^∧^
Mean ± SD (*n* = 4)	85 ± 17	0 ± 0	0 ± 0	0 ± 0	100 ± 0	78 ± 22	0 ± 0	87 ± 5	100 ± 0	70 ± 24	3 ± 5	66 ± 11
L-25/6 ^x^	25 ^b#^	0 ^b#^	0 ^b#^	0 ^b^*	100 ^x^	90 ^x^	0 ^x^	100 ^x^	100 ^∧^	80 ^x^	0 ^x^	89 ^∧^
L-15/6 ^x^	0 ^b∧^	0 ^bx^	0 ^bx^	10 ^bx^	100 ^x^	70 ^x^	0 ^x^	100 ^∧^	100 ^x^	70 ^x^	0 ^x^	100 ^∧^
L-10/6 ^x^	30 ^bx^	0 ^bx^	0 ^bx^	0 ^bx^	100 ^x^	80 ^x^	0 ^x^	100 ^x^	100 ^x^	80 ^x^	0 ^x^	70 ^x^
L-5/6 ^x^	33 ^b^^∧^	0 ^b∧^	0 ^b∧^	11 ^b^^∧^	100 ^x^	60 ^x^	0 ^x^	80 ^x^	100 ^x^	60 ^x^	0 ^x^	80 ^x^
Mean ± SD (*n* = 4)	22 ± 15	0 ± 0	0 ± 0	5 ± 6	100 ± 0	75 ± 13	0 ± 0	95 ± 10	100 ± 0	73 ± 10	0 ± 0	85 ± 13
	Mean ± SD (*n* = 8)				100 ± 0	76 ± 17	0 ± 0	91 ± 8	100 ± 0	71 ± 17	1 ± 4	76 ±15

Eight groups of 3-week-old layer chickens in Exp 2 were vaccinated twice at 4- and 6-week intervals, with 25 μg, 15 μg, 10 μg, or 5 μg of rH5-E. coli and aluminum hydroxide (alum) adjuvant. The test vaccine groups were denoted according to the chicken type (L, layer), the tested antigen dose in μg (25, 15, 10, and 5), and the time interval between doses in weeks (4 or 6). Post-vaccination sera were analyzed for the presence of anti-H5 HA antibodies using the indirect and competitive ELISAs, H5 iELISA and FluAC H5, respectively, and a hemagglutination inhibition (HI) assay with H5N2 low-pathogenic (LP) AIV. Tests were performed and interpreted as described in the Materials and Methods section. Data are presented as the percentage of chickens positive (+) in iELISA and HI tests, and positive (+) or doubtful (+/–) in the FluAC test at the indicated sampling time points. The x, ^∧^, #, and ^*^ symbols following the seropositivity percentages denote that 10, 9, 8, and 7 chickens were tested, respectively, in the group comprising 10 (x) chickens.

### Immune Responses in Broiler Chickens

Broiler chickens in Exp 1 (Table 2) responded to vaccination with 25-μg doses of alum-adjuvanted rH5-*E. coli* by production of anti-HA IgY antibodies, as detected in the H5 iELISA (Table 3). Two weeks after priming, 100% and 87.5% of chickens in the B-25/2 and B-25/4 groups, respectively, were seropositive when 1:200 serum dilutions were assayed. Four weeks post-prime, 50% of chickens in the B-25/4 group were still positive in this test. A booster vaccination, with the same antigen dose, resulted in the development of anti-H5 HA antibody responses in all chickens tested. At each of the post-boost sampling time points, 100% positivity was noted in the H5 iELISA test in the B-25/2 and B-25/4 groups. The mean endpoint titer of serum IgY antibodies against H5 HA in the B-25/2 group was ~1:28,000 at 2 weeks post-boost, while in the B-25/4 group it was ~1:129,000 at 1 week post-boost and ~1:80,000 a week later (Figure 1A). Neutralizing antibodies against H5 HA active in the FluAC H5 test and the HI assay with H5N2 LPAIV developed only in the B-25/4 group vaccinated with alum-adjuvanted rH5-*E. coli* using a 4-week prime-boost interval (Table 3). One and two weeks after the boost, 25 and 33% of chickens in this group were positive, while 12.5 and 33% were of unknown status in the FluAC H5 test, respectively. At the same time points, 25 and 37.5% of chickens were HI positive, respectively. HI antibody titers varied between 1:16 and 1:64 (Figure 1B). Analysis of the immune response pattern (Figure 1C) showed that 62.5% of chickens in the B-25/4 group developed neutralizing antibodies against H5 HA that were active in HI and/or FluAC H5 tests. Among these chickens, 37.5% were positive in both tests, while 25% were positive in the HI assay only. None of the chickens tested were exclusively FluAC H5 positive.

Immunization studies in commercial broiler chickens showed that the prime-boost vaccination schedule with alum-adjuvanted rH5-*E. coli* at a 4-week interval induced a 2.8-fold higher titer of ani-HA IgY antibodies, as measured by H5 iELISA, than the vaccination schedule with a 2-week interval (Figure 1A). More importantly, using a 4-week and not a 2-week interval between antigen doses enabled the induction of neutralizing antibody responses in the broiler chickens (Table 3). The same relationship could be observed when broiler chickens were immunized with alum-adjuvanted rH5-BEVS as the reference antigen. After the prime-boost vaccination, with a 4-week interval, all the chickens in the B-25_ref/4 group were positive in the H5 iELISA test, while 86% were positive in the B-25_ref/2 group, in which two antigen doses were administered with a 2-week delay (Table 3). In addition, the mean endpoint titer of anti-H5 HA IgY antibodies, determined for the B-25_ref/4 group at 2 weeks post-boost, was 6.7-fold higher than the endpoint titer for the B-25_ref/2 group at the same time point (Figure 1A). Antibodies active in both the FluAC H5 and HI tests were only present in the B-25_ref/4 group (Table 3), in which 75% of chickens were positive in one or both of these tests (Figure 1C).

Humoral immune responses in the B-25_ref/4 group elicited with rH5-BEVS differed from those induced in the B-25/4 group by rH5-*E. coli*, with 4.7- and 3.2-fold lower titers of anti-H5 HA IgY antibodies at 1 and 2 weeks post-boost, respectively (Figure 1A). In the B-25_ref/4 group, 50% of chickens developed neutralizing antibodies active exclusively in the FluAC H5 test (Figure 1C). The GMT of HI antibodies was 3.7 in the B-25_ref/4 group, but 8.0 in the B-25/4 group (Figure S1). However, statistically, HI titers in the B-25_ref/4 group were not significantly lower than in the B-25/4 group (*p* > 0.05; Table S1). The common finding for vaccination using alum-adjuvanted rH5-*E. coli* and rH5-BEVS was that only some of the vaccinated broiler chickens developed neutralizing antibody responses (Figure 1C). Although not satisfactory, the results from immunization studies in the commercial broiler chickens clearly indicated that the prime-boost time interval is essential for a favorable vaccination outcome. This finding is of importance for the establishment of an effective vaccination strategy against avian influenza, with the appropriate number and level of doses and adjuvant used.

### Immune Responses in Layer Chickens

The layer chickens in Exp 2 (Table 2), vaccinated twice with 5, 10, 15, or 25 μg of alum-adjuvanted rH5-*E. coli* developed anti-H5 HA antibodies following a typical pattern of primary and secondary humoral responses. After priming, a varied number of individuals in the test vaccine groups were identified as positive in the H5 iELISA (Table 4). In particular, 60–100% and 0–33% of chickens per group were seropositive when 1:200 dilutions of serum were assayed at 4 and 6 weeks post-prime, respectively. Upon re-vaccination with the same antigen doses, all tested chickens became positive in the H5 iELISA (Table 4). The endpoint titers of IgY antibodies against H5 HA in the pooled sera from particular chicken groups ranged from ~324,000 to ~848,000 1 week post-boost and from ~197,000 to ~434,000 a week later (Figures 2A,B). The respective mean values of anti-H5 HA antibody titers were 471,000 ± 182,000 and 298,000 ± 87,000.

The dynamics of antibody responses measured by the H5 iELISA were reflected in the development of neutralizing antibodies against H5 HA that were active in the FluAC H5 test and HI assay with H5N2 LPAIV (Table 4). At the post-prime time points, no chickens were FluAC H5 positive and only two chickens, each from different groups, were HI positive. One week after the boost, FluAC H5 positivity in the test vaccine groups ranged from 50 to 100%. On average, 76 ± 17% of chickens in the group were FluAC H5 positive. At the same time point, 80–100% of chickens in the groups, with an average of 91 ± 8%, were HI positive (Table 4), with GMT values of HI antibodies ranging from 1:32 to 1:69 (Figures 2C,D). Two weeks after the boost, positivity rates in the FluAC H5 test were sustained at levels ranging from 40 to 90%, with an average of 71 ± 17% (Table 4). Meanwhile, in the HI assay, positivity ranged from 56 to 100%, with an average of 76 ± 15%. At the same time point, the GMT values for HI positive sera were between 1:24 and 1:59 (Figures 2C,D).

A comparison of the results from the FluAC H5 test obtained for the different chicken groups, indicated that a 5-μg dose of alum-adjuvanted rH5-*E. coli* was the least effective in inducing antibodies active in this test. The positivity rates in the L-5/4 and L-5/6 groups, compared to the remaining groups, were 50 and 60%, respectively, vs. 70–100% at 1 week post-boost and 40 and 60%, respectively, vs. 63–90% a week later (Table 4). In contrast, no clear indication for vaccination strategy came from the HI assay results, when analyzing the overall positivity in the groups at different time points (Table 4) and the GMT values for the respective HI-positive sera (Figures 2C,D). Further analyses of data from the HI assay, that were selected as the most representative for the test vaccine groups, showed that GMT values of HI antibodies varied between 1:22 and 1:56 (Figure S2). However, no statistically significant differences in HI antibody titers were found between the groups (*p* > 0.05; Table S2). These results indicated that the induction of HI antibodies with alum-adjuvanted rH5-*E. coli* was not dependent on the applied antigen dosage or prime-boost time intervals.

A close inspection of immune response patterns (Figures 2E,F) showed that 80–100% of chickens in the test vaccine groups, with an average of 94 ± 8%, developed neutralizing antibodies against H5 HA that were active in one or both of the HI and FluAC H5 tests. The majority of these chickens were HI and FluAC H5 positive, representing 56–90% of birds in their respective groups. By comparison, 10–33% of chickens in six of the groups and none of the chickens in the other two groups were positive in the HI assay only. Exclusively FluAC H5-positive chickens constituted 10% of birds in three of the groups, but were not found in the remaining five groups. Induction of neutralizing antibody responses in all of the chickens from the test vaccine group was achieved by two immunizations with 25-μg doses of alum-adjuvanted rH5-*E. coli* at 4- and 6-week intervals or with 15- and 10-μg antigen doses given at 6-week intervals. Thus, extending the prime-boost interval from 4 to 6 weeks was beneficial for chicken vaccination, allowing a 2.5-fold reduction in the effective antigen dose. Altogether, the results using aluminum hydroxide as an adjuvant showed that the optimal scheme for vaccinating commercial layer chickens against H5N1 HPAIV is to administer two, 10-μg doses of rH5-*E. coli*, with 6 weeks between doses.

## Discussion

HPAIVs of the H5 subtype, in particular the H5N1, H5N2, H5N6, and H5N8 viruses, remain serious epidemiological concerns (1). Emerging disease outbreaks are accompanied by high virulence and mortality among domestic birds, which leads to large economic losses for the poultry industry. Moreover, H5N1 and H5N6 HPAIVs pose significant public health risks and H5N1 viruses are a permanent pandemic threat. To combat H5N1 viruses, HA-based vaccines, such as the prototype vaccine presented in this and previous (41) papers, are being developed using recombinant DNA technology. The antigen used in this study, rH5-*E. coli*, was produced in a bacterial expression system and is a non-glycosylated HA protein derived from H5N1 HPAIV. It consists of the HA1 subunit and a fragment of the HA2 subunit, with a fusion peptide and bromelain cleavage site at the N- and C-termini, respectively. Due to removal of the basic amino acids from the inter-subunit region, the protein lacks a functional cleavage site, resulting in improved stability of the protein. rH5-*E. coli* was refolded and purified from IBs, according to a refolding and purification procedure developed by our group, using two standard chromatographic beads. The purified H5 HA protein was experimentally validated in terms of conformational integrity and oligomerization status. Therefore, our design of HA protein may be used when generating antigens for other influenza vaccines. This does not require *in silico* modeling. The sequences of HAs from various influenza A virus strains can simply be determined by identification of the signal sequences, the inter-subunit, and bromelain cleavage sites.

Our bacterial HA has several unique features and presents some advantages over both the longer and shorter bacterial HAs, as discussed previously (41). rH5-*E. coli* can also be clearly distinguished from the ectodomain-based HA proteins produced in eukaryotic expression systems, despite the production of comparable fragments of viral HAs. Examples of other HA proteins produced are the 18–523-aa (H3 numbering) H5 HA proteins originating from HP (19) or LP reassortant (21) H5N1 virus strains and the 17–522-aa H1pdm09 protein (20). These truncated HA ectodomain antigens differ from our bacterial HA by the presence of N-glycans and foreign sequences, such as GCN4 isoleucine zipper trimerization motifs and Strep tags. In contrast to rH5*-E. coli*, the H5 and H1pdm09 proteins preserve the monobasic cleavage sites of the source HAs (20, 21) and therefore, are susceptible to proteolytic cleavage and low-pH fusogenic transition. Depending on the protease activity and buffer conditions, these proteins can exist as single continuous polypeptides, as the HA1 and HA2 subunits connected by a disulfide bond, or as separate HA1 and HA2 subunits. Accordingly, their conformation may switch between nonfusogenic, prefusion, and fusogenic states.

The conditions for expression and processing of eukaryotic-origin recombinant proteins and rH5*-E. coli* are completely different. The H5 HA and H1pdm09 antigens were expressed in mammalian cells with foreign signal sequences for secretion (19–21). Thus, they were produced under the same conditions as HA is produced during viral multiplication. Nevertheless, oligomerization of the proteins was achieved by intentionally adding trimerization domains. rH5-*E. coli* was expressed in a transformed *E. coli* bacterial strain without an HA signal peptide. The protein was refolded from IBs *in vitro*, using pre-determined buffer conditions. Its correct folding and oligomerization occurred in the absence of any artificial sequences. Thereby, it was shown that glycosylation is not essential to reproduce the higher-order structure of native HA antigens. However, there are data showing that the glycosylation of HA has a significant impact on the resulting antibody repertoire, by shielding antigenic sites from immune responses, and on the elicitation of neutralizing antibodies [for review, see (52)].

Despite the structural differences, the truncated HA ectodomain proteins from mammalian expression systems (19–21) and our bacterial HA, were both capable of inducing protective immune responses. Immunization of chickens or mice with H5 and ferrets with H1pdm09 HA proteins confers protection against infectious viruses and the level of protection correlates with titers of the HI (19) or HI and VN (20) antibodies. In contrast, the protection of chickens against H5N1 HPAIVs afforded by rH5-*E. coli* is correlated with positivity in the FluAC H5 test, but not with HI antibody titers, as shown in our previous paper (41). The possible mechanism of virus neutralization by antibodies active in the FluAC H5 test has been discussed in our previous report (41). The conclusion from challenge experiments was that vaccination with adjuvanted rH5-*E. coli* could provide control of H5N1 HPAIV infection and transmission rates in chicken flocks and could reduce virus shedding. Thus, the bacterial HA designed by our group is a promising alternative to the aforementioned ectodomain-based HA antigens produced at a higher cost in mammalian cells (19–21). In addition, our vaccine candidate does not raise issues related to immunogenicity and/or safety due to presence the non-viral sequences, such as the unmodified trimerization motif (53).

The aims of the immunization studies presented here were to verify the vaccine potential of our bacterial H5 HA in the two main types of chicken breeds, under semi-field conditions, and to optimize the vaccination scheme. The first, most important finding from these studies was that the prime-boost vaccination of non-SPF layer chickens with 5-μg to 25-μg doses of alum-adjuvanted rH5-*E. coli* at 4- and 6-week intervals elicited a robust antigen- and subtype-specific response, as well as HI antibody responses, against H5 HA. All of the vaccinated chickens tested developed anti-HA antibodies, as detected by H5 iELISA with a homologous H5 HA antigen (Table 4). The levels of these antibodies were high (Figures 2A,B). The majority of chickens immunized with the test vaccine were positive in the HI assay with heterologous H5N2 LPAIV and/or the FluAC H5 test, which detects antibodies against HAs of the H5-AIV subtypes (94 ± 8%; Figures 2E,F). The titers of the evoked HI antibodies (Figures 2C,D) included those considered protective (≥1:16) and those considered desirable (≥1:64) for preventing an AIV infection. The H5-subtype-specific antibodies (Table 4) reached levels observed during infection with relevant AIVs. The protective activity of these antibodies was indicated by challenge experiments using homologous and heterologous H5N1 viral strains to infect SPF, layer chickens (41). Considering the antigenic diversity of HAs from H5N1 HPAIV and H5N2 LPAIV, used as the source sequence for rH5-*E. coli* and the target antigen in the HI assay, respectively (Table 1), as well as the detectability range of the FluAC H5 test, it can be concluded that the neutralizing antibodies detected in sera of vaccinated chickens are broadly reactive with H5-subtype AIVs. It can be further hypothesized that vaccination with adjuvanted rH5-*E. coli* may confer protection against various H5Nx reassortant HPAIVs. This remains to be confirmed through challenge experiments similar to our previous studies, which have shown that rH5-*E. coli* induces inter-clade protection against H5N1 HPAIV (41).

All the chickens vaccinated with 25-, 15-, and 10-μg doses of alum-adjuvanted rH5-*E. coli* at a 6-week interval developed neutralizing anti-HA antibodies that were active in the HI and/or FluAC H5 tests (Figure 2F). Therefore, the minimum effective dose of rH5-*E. coli* was estimated as 10 μg. Since rH5-*E. coli* only partially exists as a functional oligomer (41), further reduction of this dose may be achieved by increasing the extent of antigen oligomerization. The beneficial impact of HA oligomerization on dose sparing and/or the level and quality of immune responses has been shown for HAs from both eukaryotic (18, 24) and prokaryotic (54, 55) expression systems. The minimum effective dose of rH5-*E. coli* could also be reduced by changing the inoculation route from subcutaneous to intramuscular and/or by changing the adjuvant used. Thus far, rH5-*E. coli* has only been formulated in aluminum hydroxide. Although they are safe and widely approved, aluminum compounds are not considered satisfactory, particularly when used in conventional influenza vaccines, to ensure adequate protection in weakly responding subjects (56). This has prompted the search for more potent adjuvants suitable for use in human vaccines against influenza. In the testing of recombinant H5 and/or H7 HA proteins for vaccine efficacy in chickens (19, 21–23, 25, 26, 57), alum adjuvants have rarely been used (25, 26). The adjuvants employed in these studies included commercially available Specol (19, 21), Montanide ISA 206, and ISA 70 (23), as well as an experimental adjuvant (22), all of which contained mineral oils. Except for Montanide ISA 206, which forms a water-in-oil-in-water (W/O/W) emulsion with antigens, these adjuvants were used to generate water-in-oil (W/O) emulsions, which are known to induce strong, long-term immune response (58). Consistent with the above reports, immunization studies with inactivated, whole-virus vaccines against HPAIVs have supported the use of oil-adjuvanted vaccines for AIV control in the poultry industry (59). Therefore, mineral oil-based adjuvants, such as those from the Montanide family (e.g., ISA 70 or ISA 71) should also be considered in the development of a new vaccine based on rH5-*E. coli*. Alternatively, the squalene-based (e.g., MF59 and AS03) or saponin-based (e.g., ISCOMATRIX) adjuvants may also be used, since they have been verified as compatible with influenza virus antigens (56).

The second finding from the present study was that rH5-*E. coli* formulated in aluminum hydroxide was substantially less effective when used to vaccinate broiler chickens than when used to vaccinate layer chickens. In the broiler chickens, only 62.5% positivity in the FluAC H5 and/or HI tests was achieved (Figure 1C). Although less evident owing to crucial differences in the vaccination schemes, the difference in vaccination outcomes between broiler and layer chickens was seen when testing recombinant H5 HA protein produced in *Pichia pastoris* (25, 26). To our knowledge, other recombinant H5 and/or H7 HA proteins have been evaluated for vaccination efficacy in layer chickens (19, 21, 23) or dual-purpose chickens (22, 57), but not in broiler chickens. Therefore, this is the first study comparing humoral responses of the short-lived broiler chickens and long-lived layer chickens to vaccination with adjuvanted recombinant HA antigen using a scheme that could be accomplished during the rearing period of these chickens in commercial flocks. In addition, our results highlight the need to consider the differences in immune responses between these chicken types when testing the efficacy of new vaccines for use in poultry.

The differences in outcomes of vaccination with rH5-*E. coli* between broiler and layer chickens may be related to the age of the vaccinated birds, especially at the time of priming. At this step, layer chickens were 3 weeks old, while the broiler chickens were 7 days old. Vaccination of young birds might not work optimally, since birds are not fully immunologically mature until about 3 weeks of age and maternal antibodies can interfere with vaccination (8). In line with this, the first doses of evaluated vaccines based on recombinant H5 HA proteins have typically been administered to SPF and/or non-SPF layer and dual-purpose chickens, 3 to 9 weeks after hatching (19, 21, 23, 25). SPF dual-purpose chickens were primed with bacterially produced msyB:H5 protein at 12 days of age (57), while the non-SPF broiler chickens were primed with a yeast-derived H5 HA protein at the age of 7 or 8 days (26). In turn, a baculovirus-derived H5 HA antigen has been tested by applying a single dose of the vaccine to SPF dual-purpose chickens at either 3 weeks or 1 or 2 days after hatching (22). Although less consistently than chickens vaccinated at 3 weeks of age, the chickens, free of maternal AI antibodies, vaccinated at 1 or 2 days of age, responded to vaccination by the production of HI antibodies. In some cases, the titers of these antibodies were comparable to those achieved using whole virus preparations. Susceptibility of very young chickens to vaccination is additionally confirmed by the fact that commercial vaccines are routinely given on day 1 after hatching (51). Thus, the immune status of the 7-day-old broiler chickens at priming and the interference of maternal antibodies do not seem to be the only causes of the non-satisfactory results in broiler chickens vaccinated with alum-adjuvanted rH5-*E. coli*. This can be further explained in view of results from comparative studies of the immune responses of SPF, broiler and layer chickens of the same age, when vaccinated with the model antigen, TNP-KLH (49). In that study, broiler chickens developed much weaker and shorter-lasting IgY antibody responses in comparison to layer chickens. In addition, the overall T-cell immune response in broiler chickens was lower than in layer chickens. These results indicate that different immunocompetencies may contribute appreciably to the divergent outcomes of vaccination with rH5-*E. coli* in the two types of chicken breeds.

The third finding from these immunization studies relates to the effect of the prime-boost time interval on vaccination outcome. Two vaccinations of broiler chickens with alum-adjuvanted rH5-*E. coli* at a 4-week interval was more efficient at eliciting a high titer of antibodies against HA than two vaccinations at a 2-week interval (Figure 1A). More importantly, a 4-week delay between the prime and boost injections was required to induce neutralizing antibodies that were active in the HI and/or FluAC H5 tests (Table 3). The same relationship was observed when rH5-BEVS was used as the reference antigen to immunize broiler chickens (Figure 1A, Table 3). In addition, vaccinations of layer chickens with adjuvanted rH5-*E. coli*, with a 6-week interval, were advantageous over vaccinations with a 4-week interval. This was evidenced by the 2.5-fold reduction in antigen dose needed to induce neutralizing antibodies in all of the vaccinated chickens (Figure 2F). To our knowledge, this is the first report that provides a comparison of responses in chickens vaccinated with HA-based vaccines at different inter-dose intervals. In chicken immunization studies reported to date, using recombinant HAs of various origins, a single (19, 22) or usually two (19, 21, 23, 25, 26, 57) antigen doses have been administered. Prime-boost vaccinations have been performed at one specified time interval of 2 or 3 weeks (19, 21, 23, 57). As an exception, a recombinant H5 HA protein produced in *P. pastoris* has been tested in 7-day-old broiler chickens and 3-week-old layer chickens using 2- and 4-week prime-boost intervals, respectively (25, 26). Due to differences in both the age and immunoreactivity between the vaccinated chickens, the lower vaccination efficacy observed in the broiler chickens compared to the layer chickens cannot necessarily be attributed to the shorter inter-dose delay. In contrast, results presented here clearly show that optimizing the time interval between doses of HA-based vaccines is essential for the induction of neutralizing antibody responses in chickens. Significance of the inter-dose interval for vaccination outcome has been appreciated in examinations of other vaccines for poultry. The exemplary studies were performed for vaccine against infectious bronchitis virus (60). In addition, the effect of interval between vaccinations against different viral pathogens on protection against challenge has been extensively tested [e.g., (61)]. Such studies result in recommended vaccination programs for individual poultry sectors, as presented in the Merck Veterinary Manual (62).

The prime-boost vaccination with alum-adjuvanted rH5-*E. coli* did not provide satisfactory neutralizing antibody responses in the broiler chickens, even though the optimized, 4-week interval between doses was applied (Figure 1C). However, such an immunization scheme is not reasonable in commercial chicken production, because broiler chickens are usually kept for only 6 weeks before they are slaughtered. A solution for the vaccination of the short-lived broiler chickens with rH5-*E. coli* may be the use of an immunopotentiator that would help overcome their low responsiveness and induce life-long immunity with a single vaccination. To enhance the efficacy and duration of immunity, many chicken vaccines are formulated in water-in-oil (W/O) emulsions (63). However, the use of oil-based adjuvants in vaccines for commercial broiler chickens is very limited. In many countries, the mandated withdrawal time after vaccination with an inactivated oil-adjuvanted vaccine is longer than the lifespan of the bird (8). In addition, the post-hatch vaccination of broiler chickens may not be justified because of the cost of vaccine administration. Instead, commercial broiler chicken hatcheries commonly use *in ovo* vaccination technology (51, 63). A number of agents, including inactivated avian influenza oil-emulsion vaccines and vectored vaccines encoding H5 or H7 AIV HAs, have been shown to induce immune responses following *in ovo* administration (63). These agents do not comprise recombinant HA proteins. Therefore, our subunit vaccine is unlikely to be an option for broiler chickens, owing to their short lifespan. In contrast, the slower-growing meat birds (e.g., Chinese yellow-feathered chickens), which are not marketed until they are ~90 days old, and the broiler breeders that are kept for up to 2 years (8) may be suitable targets for immunization with rH5-*E. coli*. For this purpose, the antigen and/or vaccine composition will need to be improved and the vaccination scheme will need to be further optimized.

Two doses of alum-adjuvanted rH5-*E. coli* were necessary, but sufficient, to induce a high level of neutralizing antibodies in laying chickens (Figures 2E,F). In this type of chicken, a prime-boost vaccination scheme is feasible, due to their 2-year lifespan in both breeding and commercial flocks. A booster vaccination, aimed at maintaining the antibody response, is also common practice in long-lived poultry species (51, 63). The semi-field efficacy trials presented here and the previously reported laboratory challenge experiments (41) in non-SPF and SPF layer chickens, respectively, confirm the vaccine potential of rH5-*E. coli*. Thus, rH5-*E. coli* is a promising candidate for a vaccine against H5N1 HPAIV and possibly other H5Nx viruses. According to the poultry AIV vaccination recommendations (8), the target for immunization with rH5-*E. coli* would be table egg layer chickens and breeders. As much of the focus on vaccine technology for AIV is directed at producing human vaccines for AIV in the event of a pandemic (8), our future research will expand to the development of a vaccine for humans.

## Data Availability

All datasets generated for this study are included in the manuscript and/or the Supplementary Files.

## Ethics Statement

The experiments in this study were approved by the Second Local Ethical Committee for Animal Experiments at the Medical University of Warsaw (Poland), Permit Number 17/2009. All efforts were made to minimize suffering of the animals.

## Author Contributions

VS conceived and designed the experiments, analyzed the data, prepared the tables and figures, and wrote the manuscript. AR-C, NŁ, and IS produced the vaccine antigen. AR-C participated in chicken vaccinations. KF performed ELISAs and statistical analyses. VC-A performed the hemagglutination assays and assisted with data visualization. MK-B prepared the genetic constructs. GP contributed to funding acquisition. All the authors have read and approved the final manuscript.

### Conflict of Interest Statement

The results presented in this work are contained in Patent Applications P.408649 (2014-06-24), PCT/PL2015/050025 (2015-06-24) and U.S. Application No. 15/320,484 (2016-12-20, decision pending). The authors declare that the research was conducted in the absence of any commercial or financial relationships that could be construed as a potential conflict of interest.
