# Supplementary material for: Prime-Boost Vaccination With a Novel Hemagglutinin Protein Produced in Bacteria Induces Neutralizing Antibody Responses Against H5-Subtype Influenza Viruses in Commercial Chickens
Source: Front Immunol. 2019 Sep 4;10:2006. doi: 10.3389/fimmu.2019.02006 (PMC6736996; doi:10.3389/fimmu.2019.02006)
Supplement: Supplementary file 1 [file Data_Sheet_1.pdf]

Supplementary Material

Prime-Boost Vaccination with a Novel Hemagglutinin Protein Produced in Bacteria Induces Neutralizing Antibody Responses Against H5-Subtype Influenza Viruses in Commercial Chickens

Violetta Sączyńska*, Agnieszka Romanik-Chruścielewska, Katarzyna Florys, Violetta Cecuda-Adamczewska, Natalia Łukasiewicz, Iwona Sokołowska, Małgorzata Kęsik-Brodacka, Grażyna Płucienniczak

* **Correspondence:** Violetta Sączyńska: saczynskav@iba.waw.pl

Chicken Responses to Vaccination with Hemagglutination-Inhibiting Antibodies

Broiler and layer chickens were vaccinated with the test and/or reference H5 hemagglutinin (HA) antigens, rH5-*E. coli* and rH5-BEVS, respectively, in the presence of aluminum hydroxide (alum) adjuvant. rH5-*E. coli* (aa 17–522, ΔRRRKKR) was produced in a bacterial expression system, while rH5-BEVS (aa 17–530, ΔRRRKKR, 6x His) was produced in a baculovirus expression vector system. The test vaccine groups were denoted according to the chicken type (B, broiler or L, layer), the test or reference antigen dose in µg (25 or 25_ref, 15, 10, and 5), and the time interval between doses in weeks (2, 4, and 6). Sera collected during the time-course of vaccinations were analyzed using a hemagglutination inhibition (HI) test with heterologous, A/turk/Italy/80(H5N2) low-pathogenic (LP) AIV, at a HI unit (HIU) of 1:8, using specific pathogen-free (SPF) chicken erythrocytes. The HI titer was defined as the reciprocal of the highest dilution of serum that caused an inhibition of hemagglutination activity with 4 hemagglutination units (HAU) of the inactivated antigen. Serum HI titers equal to or greater than 1:16 were considered positive, while those equal or lower than the detection limit (1:8), were considered negative. Sera with undetectable HI antibodies were assigned a value of 1.0.

The HI test results from each sampling time point are presented in the manuscript. Here, the highest HI antibody titers determined for each of the HI-positive and HI-negative chickens (values ≥ 16 and 8.0 or 1.0, respectively) were selected to compare HI antibody responses between the test vaccine groups. These data were used in the geometric mean titer (GMT) calculations and in statistical analyses. Kruskal-Wallis and Mann-Whitney U non-parametric tests for comparisons of multiple or two groups, respectively, were performed using Statistica software (StatSoft, Cracow, Poland). A value of $p < 0.05$ was considered significant.

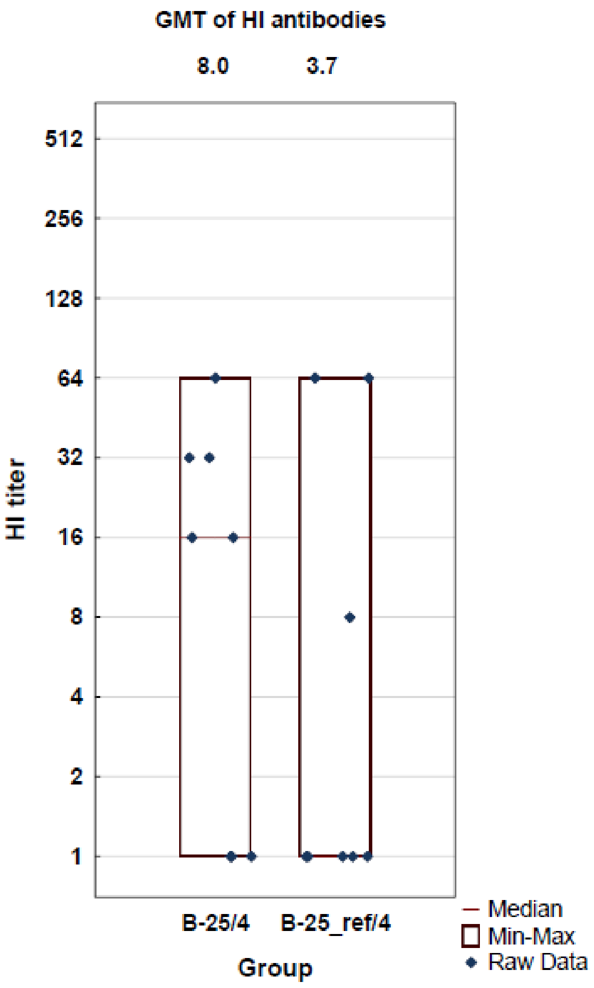

Supplementary Figure S1. HI antibodies in sera from broiler chickens vaccinated with alum-adjuvanted rH5-*E. coli* or rH5-BEVS

Two groups of 1-week-old broiler chickens (8 per group) from Exp 1, denoted B-25/4 and B-25_ref/4, were vaccinated twice at a 4-week interval with 25 µg of rH5-*E. coli* or rH5-BEVS, respectively. HI tests were performed for sera collected post-prime and post-boost. Raw data represent the highest HI antibody titers determined for individual chickens during the post-vaccination period. Results for particular groups are summarized as minimum and maximum values, medians, and GMT values.

Supplementary Table S1. A comparison of HI antibody responses between broiler chickens vaccinated with alum-adjuvanted rH5-*E. coli* or rH5-BEVS

	Mann-Whitney U Test (B, 2 groups)<br>By variable: group<br>Marked tests are significant at p <.05000									
Variable	Rank Sum B-25/4	Rank Sum B-25_ref/4	U	Z	p-value	Z adjusted	p-value	Valid N B-25/4	Valid N B-25_ref/4	2*1 sided exact p
HI titer	74.50000	61.50000	25.50000	0.630126	0.528613	0.676481	0.498736	8	8	0.505361

The data presented in Figure S1 were analyzed using the Mann-Whitney U test and the results are presented here. HI antibody titers in the B-25/4 group were not significantly higher than those in the B-25_ref/4 group ($p > 0.05$).

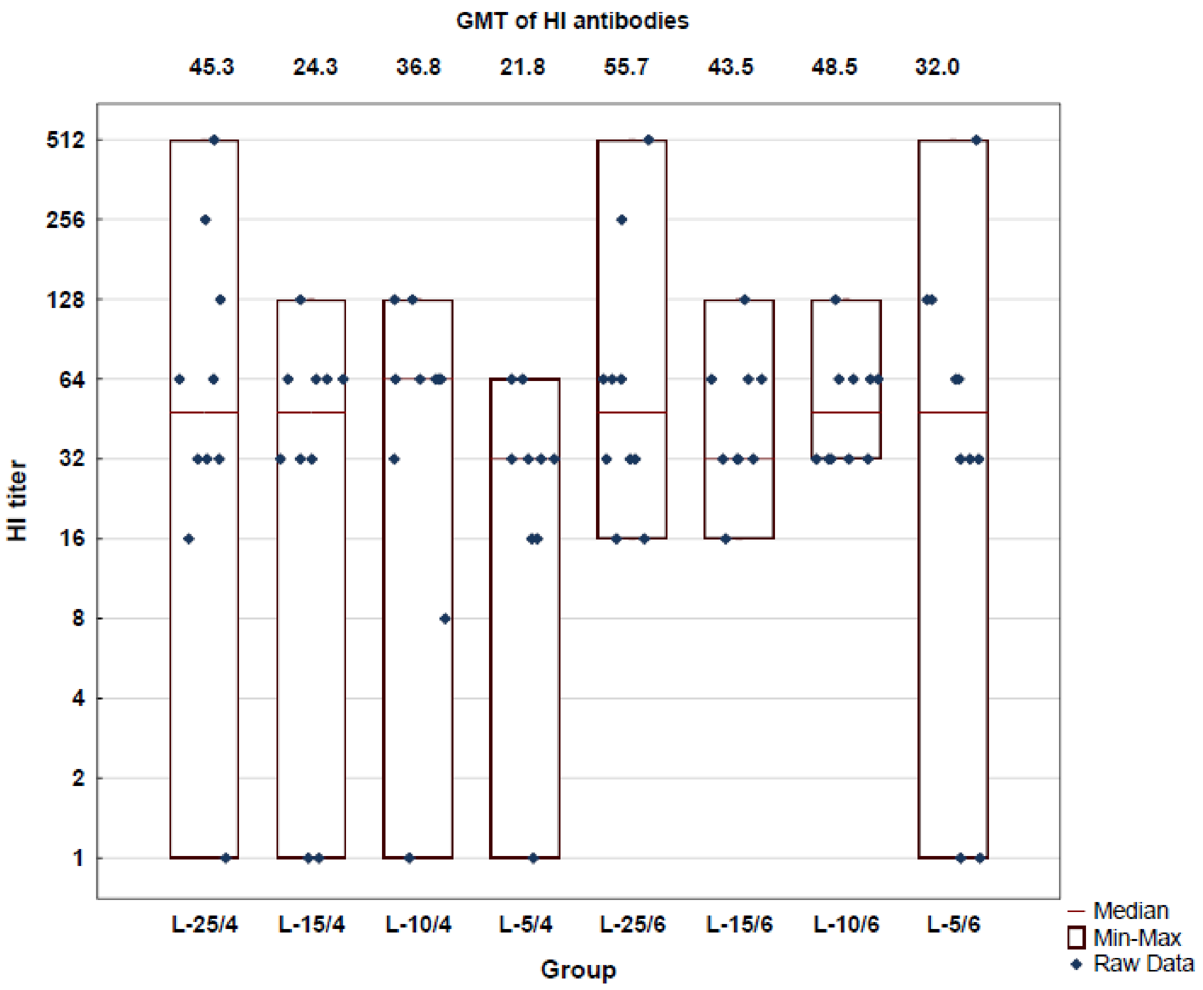

Supplementary Figure S2. HI antibodies in sera from layer chickens vaccinated with alum-adjuvanted rH5-*E. coli*

Eight groups of 3-week-old layer chickens (10 per group) from Exp 2, denoted L-25/4, L-15/4, L-10/4, L-5/4, L-25/6, L-15/6, L-10/6, and L-5/6, were vaccinated twice with 25 µg, 15 µg, 10 µg, or 5 µg of rH5-*E. coli* at 4- or 6-week intervals. HI tests were performed for sera collected post-prime and post-boost. Raw data represent the highest HI antibody titers determined for individual chickens during the post-vaccination period. Results for particular groups are summarized as minimum and maximum values, medians, and GMT values.

Supplementary Table S2. A comparison of HI antibody responses between the layer chicken groups vaccinated with alum-adjuvanted rH5-*E. coli*

	Kruskal-Wallis ANOVA by Ranks; HI titer (L, 8 groups)<br>Independent (grouping) variable: group<br>Kruskal-Wallis test: H (7, N= 78) =3.975032 p =.7826			
Dependent:<br>HI titer	Code	Valid N	Sum of Ranks	Mean of Ranks
L-25/4	101	10	422.0000	42.20000
L-15/4	102	10	373.0000	37.30000
L-10/4	103	10	444.0000	44.40000
L-5/4	104	9	243.5000	27.05556
L-25/6	105	10	416.0000	41.60000
L-15/6	106	9	350.0000	38.88889
L-10/6	107	10	419.0000	41.90000
L-5/6	108	10	413.5000	41.35000

The data presented in Figure S2 were analyzed using the Kruskal-Wallis test, and the results are presented here. No significant differences in HI antibody titers were found between the groups of vaccinated layer chickens ($p > 0.05$).
